# Knowledge and Perceptions of Oncology Care Among Non-oncology Clinicians: A Cross-Sectional Survey

**DOI:** 10.7759/cureus.117675

**Published:** 2026-10-09

**Authors:** Nishitha Shetty, Santhosh Kumar Devadas, Jason Fernandes Joekim, Kiran Kumar BR

**Affiliations:** 1 Medical Oncology, Father Muller Medical College Hospital, Mangaluru, IND; 2 Medical Oncology, Ramaiah Medical College, Bengaluru, IND

**Keywords:** cancer knowledge and attitudes, continuing medical education, cross-sectional survey, india, non-oncologist physicians, oncology, physician–oncologist communication

## Abstract

Background: Non-oncologist clinicians are frequently the first point of contact for patients with cancer; however, their knowledge, attitudes, and referral behavior toward oncology services are incompletely characterized, particularly in medical colleges and private and government hospital networks serving non-metropolitan populations.

Methods: A cross-sectional survey was conducted among 101 non-oncologist clinicians practicing in India, utilizing a 26-item structured questionnaire administered electronically. Categorical variables were presented as frequencies and percentages. The single quantitative item, which assessed the perceived benefit of aggressive cancer treatment on a scale from 0 to 10, was reported as mean ± standard deviation (SD) and median with interquartile range (IQR). Associations were evaluated using Pearson's chi-square test, with Monte Carlo permutation correction applied when necessary, and Cramér's V. Differences between groups for the quantitative item were analyzed using the Mann-Whitney U test or the Kruskal-Wallis H test.

Results: Most respondents, 91 (90.1%), had no formal oncology training, yet 63 (62.4%) encountered cancer patients at least weekly, and 88 (87.1%) were not fully comfortable managing cancer-related symptoms without an oncology referral. Attitudes toward oncologists (94; 93.1% “essential in cancer care”) and palliative care (100; 99.0% agreement on benefit) were favorable, while only 16 (15.8%) felt “very familiar” with newer cancer therapies. Patient preference against referral 42 (41.6%) and cost 31 (30.7%) were the leading barriers. Practice setting was associated with communication quality with oncologists (χ² = 18.04, df = 8, p = 0.021, Cramér's V = 0.30), and region of practice with consistency of oncologist involvement in long-term care (χ² = 16.75, df = 8, p = 0.033, V = 0.29); formal training was not associated with any outcome.

Conclusions: Healthcare professionals generally have a positive attitude toward oncology and palliative care; however, many lack formal training. Communication and continuity of care also remain challenging, mainly because of differences in healthcare settings and locations rather than individual training. Regular oncology education and simple, standardized communication systems between healthcare providers could help improve cancer care, especially in centers without dedicated oncology services.

## Introduction

Cancer remains a leading cause of global morbidity and mortality, with an increasing burden in resource-constrained settings worldwide. The Global Cancer Observatory (GLOBOCAN) estimates approximately 20.6 million new cancer cases and 9.8 million cancer deaths worldwide in 2024, with projections of approximately 34 million annual cases by 2050 [1]. This growth is expected to be greatest in low- and middle-income countries, where disparities in healthcare infrastructure, oncology workforce, and access to evidence-based therapies remain significant challenges in cancer treatment [2]. Non-oncologist clinicians consult with patients at multiple points across the cancer care trajectory-at presentation, during treatment complications, in survivorship, and at the end of life-and often act as gatekeepers to oncology referral; therefore, their knowledge, attitudes, and communication practices materially influence how quickly patients receive definitive care.

Across diverse healthcare settings, survey-based studies consistently show limited oncology knowledge among non-oncologist physicians, including hospitalists reporting low confidence in managing oncology-related issues [3], internal medicine and intensive care physicians reporting persistent terminology and communication gaps [4], and emergency medicine data describing shortfalls in cancer therapeutics and oncologic emergencies [5]. Inadequate oncology exposure during training has been linked to similar knowledge gaps among early-career physicians [6,7]. Structural factors also limit timely access to oncological care. Studies from rural India describe delays due to low awareness, stigma, inadequate screening, and financial barriers affecting patients and providers alike [8], and similar cost, logistical, and cultural barriers have been reported among women seeking breast cancer treatment [9]. Shared-care literature further identifies unclear roles and limited communication as barriers to coordination with oncology services [10,11], while multidisciplinary tumor boards and integrated palliative care models have improved treatment coordination and outcomes, underscoring the need for stronger links between non-oncologist clinicians and oncology teams [12-14].

Attitudes toward oncologists add another dimension to this picture. Early scenario-based surveys found that non-oncology physicians often endorsed treatment approaches that lagged behind then-current oncologic standards [15-17], and more recent multicenter data from Pakistan similarly describe mixed attitudes toward cancer treatment and palliative care, including skepticism about chemotherapy tolerability [18]. Such attitudes-trust in oncologists' expertise, confidence in referral decisions, and beliefs about treatment benefits-may shape engagement with cancer care independently of formal knowledge and merit direct assessment alongside knowledge and structural barriers.

Few studies have comprehensively evaluated non-oncologist clinicians' knowledge, perceptions, and referral practices regarding cancer care in an integrated manner. This cross-sectional survey of clinicians from medical colleges, private, and government hospitals in India assessed professional characteristics, cancer care experience, treatment beliefs, referral patterns, oncology communication, and factors influencing these outcomes.

## Materials and methods

This was a questionnaire-based, cross-sectional survey of licensed non-oncologist clinicians practicing in medical colleges (52.5%), private hospitals (34.7%), and government hospitals (12.9%) across non-metropolitan (62.4%), metropolitan (26.7%), and rural (10.9%) areas in India. The study was conducted between August 2025 and March 2026. Clinicians were recruited through departmental circulation and professional networks and invited to complete the survey. The survey was administered electronically using Google Forms (Google LLC, Mountain View, CA, USA). Eligible participants were registered medical practitioners actively engaged in clinical practice who were neither formally certified nor undergoing training in medical, radiation, or surgical oncology. These participants were classified as non-oncology physicians. The questionnaire further assessed prior structured oncology exposure during medical training, defined as an oncology posting or rotation of approximately one month during residency without subsequent specialization in oncology. Participants with such prior exposure were categorized as having prior structured oncology exposure during training, while those without such exposure were classified as non-oncology physicians without formal oncology exposure.

Board-certified oncologists, oncology trainees (e.g., DM/fellowship trainees in oncology), and respondents with incomplete questionnaires were excluded from the study. The inclusion and exclusion criteria are summarized in Table 1. The participants included clinicians from medicine and allied specialties (66.4%), surgical specialties (27.7%), and general practice/other specialties (6.0%), reflecting a diverse group of non-oncologist healthcare professionals.

**Table 1:** Inclusion and exclusion criteria for study participation DM: Doctor of Medicine

Criterion type	Criterion
Inclusion	Registered medical practitioner currently in active clinical practice
Inclusion	Not formally trained in medical, radiation, or surgical oncology
Exclusion	Board-certified oncologists, oncology trainees (e.g., DM/fellowship trainees in oncology)
Exclusion	Oncology trainees
Exclusion	Respondents with incomplete questionnaires

Questionnaire

The investigator-developed questionnaire comprised 26 items covering four domains: (1) demographic and professional characteristics; (2) the frequency of and self-reported comfort with cancer-related clinical encounters; (3) knowledge and perceptions regarding cancer, chemotherapy, oncologists, and palliative care; and (4) referral practices and interactions with oncology services. The questionnaire also included three optional free-text questions. These responses were summarized by response rate only and were not subjected to qualitative content analysis; therefore, they are not further reported in this study. The complete questionnaire is provided in the Appendices. The questionnaire was developed by the investigator team based on domains identified from the existing literature and was reviewed by the co-authors for face validity, relevance, and clarity. It was subsequently piloted among a small group of clinicians prior to full deployment, and minor refinements were made based on the pilot feedback.

Statistical analysis

Categorical variables were summarized as frequencies and percentages of the total sample (N = 101). Multiple-response items, in which respondents could select more than one option, are reported as the number and percentage of the 101 respondents endorsing each option; therefore, the percentages sum to more than 100%. The single quantitative item (perceived benefit of aggressive cancer treatment, 0-10) was summarized as mean ± SD and median (IQR), with the Shapiro-Wilk test used to assess normality of distribution. Two optional free-text items were summarized only by their response rates.

Associations between demographic/professional characteristics and outcomes were tested using Pearson's chi-square test, with Fisher's exact test for 2×2 tables and an exact Monte Carlo permutation p-value (20,000 resamples) reported alongside the asymptotic value, where more than 20% of the cells had an expected count of < 5. Effect sizes were reported as Cramér's V (<0.10, negligible; 0.10-0.29, small; 0.30-0.49, moderate; and ≥0.50, large). Because the quantitative item was non-normally distributed (Shapiro-Wilk p < 0.001), group comparisons were performed using the Mann-Whitney U test (two groups) or the Kruskal-Wallis H test (three or more groups). A two-tailed α = 0.05 was used; given that approximately 20 associations were tested, findings with 0.01 < p < 0.05 are reported as hypothesis-generating rather than confirmatory (a Bonferroni-corrected threshold for ~20 comparisons would require p < 0.0025 for family-wise significance). Analyses were performed using Python 3 (Python Software Foundation, Wilmington, DE, USA), Pandas, and SciPy (NumFOCUS, Austin, TX, USA).

## Results

Sample characteristics

The sample was predominantly aged 30-39 years (31, 30.7%) and male (65, 64.4%) (Table 2). The years in clinical practice were fairly evenly distributed (24, 23.8% <5 years; 20, 19.8% 5-10 years; 29, 28.7% 11-20 years; and 28, 27.7% >20 years). Just over half of the respondents practiced in medical college settings (53, 52.5%), and most of them practiced in non-metropolitan cities (63, 62.4%). Most respondents (91, 90.1%) reported no prior structured oncology exposure; the remaining 10 (9.9%) reported brief oncology exposure such as a compulsory oncology posting during residency, not formal oncology specialization.

**Table 2:** Demographic and professional characteristics of respondents (N = 101)

Characteristic	n	%
Age <30 years	24	23.8
Age 30–39 years	31	30.7
Age 40–49 years	19	18.8
Age 50–59 years	17	16.8
Age ≥60 years	10	9.9
Male	65	64.4
Female	36	35.6
Practice <5 years	24	23.8
Practice 5–10 years	20	19.8
Practice 11–20 years	29	28.7
Practice >20 years	28	27.7
Medicine & allied specialties	67	66.4
Surgical specialties	28	27.7
General practitioners	06	5.9
Medical college	53	52.5
Private hospital	35	34.7
Government hospital	13	12.9
Non-metropolitan city	63	62.4
Metropolitan city	27	26.7
Rural area	11	10.9
No prior oncology training exposure	91	90.1
Prior oncology training exposure (e.g., brief residency posting; none board-certified)	10	9.9

Cancer-related clinical exposure and confidence in care

Cancer encounters were frequent among respondents, with 63 (62.4%) reporting contact with at least one cancer patient per week. Half of the clinicians (51, 50.5%) reported encountering both early- and advanced-stage presentations, while 37 (36.6%) reported predominantly advanced-stage disease. Confidence in managing cancer-related symptoms without referral was limited; 88 (87.1%) of the respondents were either uncomfortable or managed only selected symptoms before referral (Table 3). Comfort disclosing a cancer diagnosis to patients was more favorable: 40 (39.6%) felt confident disclosing a diagnosis themselves, a further 43 (42.6%) were comfortable depending on the clinical situation, and only 18 (17.8%) preferred that an oncologist handle disclosure; thus, most respondents were willing to take at least some role in diagnosis disclosure (Table 3). This did not differ significantly by training status, years in practice, practice setting, or sex.

**Table 3:** Cancer-related clinical exposure and confidence in care

Parameter	n (%)
Frequency of seeing cancer patients	
<1 patient/week	38 (37.6)
1–5 patients/week	38 (37.6)
>5 patients/week	25 (24.8)
Perceived stage at presentation	
Predominantly early-stage	13 (12.9)
Predominantly advanced-stage	37 (36.6)
Both early and advanced stages	51 (50.5)
Comfort managing cancer-related symptoms without referral	
Comfortable with all symptoms	13 (12.9)
Comfortable with some symptoms; refers others	52 (51.5)
Not comfortable	36 (35.6)
Comfort disclosing a cancer diagnosis to patients	
Confident in disclosing	40 (39.6)
Depends on the clinical situation	43 (42.6)
Prefer oncologist to disclose	18 (17.8)

Knowledge, perceptions, and beliefs regarding oncology care

Overall, the perceptions of cancer care were positive in this study. More than half of the respondents (59, 58.4%) believed that cancer is curable in many cases, and 100 (99.0%) agreed that palliative care provides meaningful benefits to patients. However, familiarity with newer cancer therapies was limited, with only 16 (15.8%) of respondents reporting being very familiar with these. The perceived benefit of aggressive cancer treatment was high (median score 8.0, IQR 7.0-9.0), demonstrating a ceiling effect in the responses (Table 4).

**Table 4:** Knowledge, perceptions, and beliefs regarding oncology care

Parameter	n (%)
Cancer curability	
Believed cancer is curable in many cases	59 (58.4)
Oncologists consider quality of life in decisions	
Agreed/strongly agreed	52 (51.5)
Perceived benefit of palliative care	
Agreed/strongly agreed	100 (99.0)
Familiarity with newer cancer therapies	
Very familiar	16 (15.8)
Limited familiarity/only heard of them	77 (76.2)
Unfamiliar	08 (7.9)

Perceptions of chemotherapy and oncologists

Chemotherapy was most commonly perceived as curative in selected cancers (83, 82.2%) and as a means of prolonging survival (78, 77.2%). Respondents viewed oncologists favorably, with most considering them essential to cancer care (94, 93.1%) and evidence-based practitioners (77, 76.2%). However, 42 (41.6%) of respondents noted variability in the quality of communication with oncology services, while negative perceptions of oncologists were uncommon (≤3, ≤3.0%) (Table 5).

**Table 5:** Perceptions of chemotherapy and oncologists

Perception	n (%)
Chemotherapy	
Curative in some cancers	83 (82.2)
Can prolong survival	78 (77.2)
Oncologists	
Essential in cancer care	94 (93.1)
Follow evidence-based approaches	77 (76.2)
Communication quality varies	42 (41.6)
Negative perceptions (profit-driven/toxic dosing)	≤3 (≤3.0)

Referral practices and interaction with oncology services

Barriers to oncology referral were primarily related to patient and healthcare system factors rather than clinician attitudes. Patient reluctance (42, 41.6%), cost concerns (31, 30.7%), and poor patient follow-up (23, 22.8%) were the most frequently reported barriers to referral. While 60 (59.4%) of respondents considered communication with oncology services to be highly collaborative, 28 (27.7%) reported occasional, minimal, or no communication, and a further 28 (27.7%) reported that the oncologist rarely or never involved them in ongoing patient care (Table 6).

**Table 6:** Barriers to oncology referral and care coordination

Parameter	n (%)
Reasons for not referring patients to oncology	
Patient preference against referral	42 (41.6)
Cost concerns	31 (30.7)
Poor patient follow-up	23 (22.8)
Perception that “nothing more can be done”	23 (22.8)
Overemphasis on chemotherapy	6 (5.9)
Cultural beliefs	6 (5.9)
Patients dissatisfied with oncology care encountered	
Yes	35 (34.7)
Communication with oncologists	
Very collaborative	60 (59.4)
Occasional/minimal/no communication	28 (27.7)
Oncologist involvement of referring clinician in long-term care	
Rarely/not at all involved	28 (27.7)

Inferential associations

Approximately 20 associations between clinician and practice characteristics and the prespecified outcomes were evaluated. Given the exploratory nature of the survey and the sample size of 101 participants, these analyses were undertaken a priori to explore the breadth of potential correlates and were considered hypothesis-generating rather than confirmatory. Prior formal oncology exposure was reported by 10 participants and was not significantly associated with any of the assessed outcomes, including comfort in symptom management, comfort in communicating cancer diagnoses, perceptions of cancer curability, familiarity with newer cancer therapies, or interactions with oncologists (all p ≥ 0.11). Similarly, years in clinical practice and sex were not significantly associated with any of the assessed outcomes (all p ≥ 0.11). Given the exploratory design, sample size, and multiple comparisons, these findings should be interpreted as hypothesis-generating and require confirmation in larger studies.

Two associations reached nominal significance (Table 7), both confirmed by Monte Carlo permutation testing: practice setting with quality of communication with oncologists and region of practice with consistency of oncologist involvement in the long-term care. Clinicians in private hospitals most often reported “very collaborative” communication, while government-hospital clinicians reported proportionally more “minimal” communication. Rural-area clinicians reported more variable or inconsistent oncologist involvement than metropolitan or non-metropolitan city clinicians. Neither finding met the Bonferroni-corrected threshold for ~20 comparisons (p < 0.0025), and both should be regarded as hypothesis-generating.

**Table 7:** Statistically significant associations between practice characteristics and referral-related outcomes (N = 101)

Predictor	Outcome	χ² (df)	p (asymptotic)	p (Monte Carlo)	Cramér's V
Practice setting	Communication with oncologists	18.04 (8)	0.021	0.020	0.30 (moderate)
Region of practice	Oncologist involvement in long-term care	16.75 (8)	0.033	0.033	0.29 (small–moderate)

For the quantitative item (perceived benefit of aggressive treatment), no grouping variable showed a significant difference: prior oncology training exposure (Mann-Whitney U = 507.5, p = 0.545), years in practice (Kruskal-Wallis H = 4.16, p = 0.244), practice setting (H = 1.45, p = 0.484), or sex (H = 1.76, p = 0.185).

## Discussion

This cross-sectional survey of 101 non-oncologist clinicians across medical colleges, private, and government hospital settings in India provides an integrated picture of how clinicians outside oncology perceive, engage with, and refer patients for cancer care in India. Three findings stand out from this study.

First, prior structured oncology exposure during training was rare (10, 9.9%, and limited to brief postings rather than formal specialization) despite frequent clinical contact, and comfort in managing cancer-related symptoms without referral was limited. This echoes international survey data in which non-oncologist physicians consistently rate their own oncology knowledge as outdated or insufficient, from hospitalists [3], to internal medicine and intensive care physicians [4], to emergency medicine data describing shortfalls in cancer therapeutics and oncologic emergencies [5]. Low familiarity with newer cancer therapies (16, 15.8% “very familiar”) mirrors India-specific gaps in domains such as fertility preservation referral pathways [7]. Formal training was not statistically associated with any measured outcome, although the small trained subgroup (n = 10) limited statistical power; this should not be interpreted as evidence that training does not matter, particularly as limited oncology exposure during training is itself thought to constrain the future workforce [6].

Second, attitudes toward oncologists and palliative care were largely positive. Most participants considered oncologists essential for cancer care (94, 93.1%) and agreed that palliative care benefits patients (100, 99.0%). However, only 52 (51.5%) felt that oncologists adequately considered quality of life, and 42 (41.6%) reported variability in communication with oncology services. This suggests that while oncologists are highly respected, communication and quality-of-life discussions remain inconsistent. Qualitative studies similarly report uncertainty among oncologists about their roles in shared and survivorship care, suggesting that positive attitudes alone may not ensure effective collaboration [16]. When referrals did not occur, patient preference and financial constraints were the main barriers, rather than negative clinician attitudes, consistent with studies identifying cost, low awareness, and stigma as major barriers to timely cancer care in India [8,9].

Diagnosis disclosure showed a generally positive pattern: 40 (39.6%) of respondents were confident in disclosing a cancer diagnosis themselves, while 43 (42.6%) were comfortable depending on the situation. Only 18 (17.8%) preferred that the oncologist disclose it. This suggests that most non-oncologist clinicians were willing to take some role in diagnosis disclosure. There was no significant difference based on training, years of practice, setting, or sex, suggesting that this attitude was broadly shared. This is encouraging compared with reports from Indian clinical settings, where referring doctors often leave diagnosis disclosure to oncologists because of time constraints, limited training, or family-centered communication practices [19]. In our study, reliance on the oncologist for disclosure was not the usual approach.

Third, communication and continuity of care appeared to depend more on practice setting and region than on individual clinician factors such as training, experience, or gender. This is consistent with the shared-care literature, which identifies unclear roles and lack of clear protocols as key barriers to effective collaboration [10]. Interviews from a randomized shared-care survivorship trial also highlighted the importance of two-way communication and clear roles [11]. Structured systems such as multidisciplinary tumor boards can improve treatment coordination and outcomes [12,14]. Therefore, interventions should focus on standardized referral summaries, clear points of contact, and regular feedback, rather than education alone.

Taken together, these findings suggest a low-cost agenda: targeted continuing oncology education delivered through accessible, case-based formats, consistent with the demand reported in comparable surveys of non-oncologist physicians [4]; explicit attention to patient-level and cost barriers, which our data suggest are more consequential than clinician attitudes; and structured, bidirectional communication mechanisms between referring clinicians and oncology services, prioritized in government hospitals and rural settings, where gaps were most pronounced.

This study has some limitations. It used a convenience sample recruited through an electronic platform, and the sampling frame was not available, limiting assessment of how representative the sample was. Self-reported responses may also be affected by social desirability bias, particularly regarding views of oncologists and palliative care. Additionally, the cross-sectional design limits the ability to establish causal relationships.

## Conclusions

In this cross-sectional survey, perceptions of oncologists and palliative care were favorable, while non-referral was mainly driven by financial barriers and patient preference. Communication and continuity of care varied by practice setting and region, highlighting the need for systemic solutions to address these issues. Strengthening oncology education, addressing referral barriers, and establishing structured communication pathways between non-oncologists and oncology services are practical priorities for improving the coordination and quality of cancer care beyond specialized oncology centers.

## Appendices

Study questionnaire

Knowledge and Perceptions of Oncology Care Among Non-oncology Clinicians: A Cross-Sectional Survey

Survey Instrument

Introduction: You are invited to participate in a survey exploring the perceptions of non-oncologist clinicians regarding the role of oncologists and cancer management. This survey is completely anonymous. No names, contact details, or identifying information will be collected at any stage. By proceeding with the survey, you indicate your consent to participate.

**Table 8:** Study questionnaire

Q#	Question	Response options				
Participant characteristics						
1	Age	☐ <30	☐ 30–39	☐ 40–49	☐ 50–59	☐ >60
2	Gender	☐ Male	☐ Female	☐ Prefer not to say		
3	Years in clinical practice	☐ <5	☐ 5–10	☐ 11–20	☐ >20	
4	Specialty	________________________________________________________				
5	Practice setting	☐ Government hospital	☐ Private hospital	☐ Medical college	☐ Primary health centre	☐ Rural clinic
6	Region of practice	☐ Metropolitan city	☐ Non-metropolitan city	☐ Rural area		
7	Formal exposure in oncology? (Clinical Postings/rotations-minimum of 1 month)	☐ Yes	☐ No			
Experience with cancer patients						
8	How often do you encounter cancer patients?	☐ >10 per week	☐ 5–10 per week	☐ 1–5 per week	☐ <1 per week	☐ None
9	Most common stage at first presentation in your practice	☐ Mostly early-stage	☐ Predominantly advanced-stage	☐ Both are equally common		
10	Comfort in managing cancer-related symptoms	☐ Comfortable managing most	☐ Comfortable managing some, refer others	☐ Not comfortable, usually refer		
11	Comfort in disclosing a cancer diagnosis to patients	☐ Confident in disclosing	☐ Prefer oncologist to disclose	☐ Depends on the situation		
Knowledge and beliefs						
12	Do you believe cancer is curable?	☐ Yes, many cases	☐ Only in selected cases	☐ No	☐ Unsure	
13	Your perception of chemotherapy (select all that apply)	☐ Curative in some cancers	☐ Can prolong survival	☐ Toxic	☐ Expensive	☐ Not suitable for elderly patients
		☐ One of several treatment modalities	☐ Reduces quality of life			
14	Your perception of oncologists (select all that apply)	☐ Essential in cancer care	☐ Evidence-based approach	☐ Primarily focus on drug therapy	☐ Good communication skills	☐ Communication quality varies
		☐ Ethical in practice	☐ Profit-driven	☐ Prescribe high-end expensive drugs	☐ Prescribe toxic doses of chemotherapy	☐ Unsure
15	Aggressive cancer treatment provides meaningful clinical benefit in appropriately selected patients	☐ 0 – Strongly disagree	☐ 1	☐ 2	☐ 3	☐ 4
		☐ 5	☐ 6	☐ 7	☐ 8	☐ 9
		☐ 10 – Strongly agree				
16	Do oncologists consider quality of life in decision-making?	☐ Always	☐ Sometimes	☐ Unsure		
17	Perceived benefit of palliative care	☐ Strongly agree	☐ Agree	☐ Disagree	☐ Strongly disagree	
18	Familiarity with newer cancer therapies (e.g., immunotherapy, targeted therapy, CAR-T therapy)	☐ Very familiar	☐ Somewhat familiar	☐ Only heard of them	☐ Not familiar	
Referral practices						
19	Reasons for not referring patients to oncology services (select all that apply)	☐ Cost concerns	☐ Perception that nothing more can be done	☐ Fear of treatment-related toxicity	☐ Poor patient follow-up	☐ Limited access to oncology services
		☐ Patient preference against referral	☐ Cultural beliefs	☐ Concern about losing patient follow-up	☐ Perceived overemphasis on chemotherapy	☐ Viewed as waste of time, energy, or resources
20	Have you encountered patients dissatisfied with oncology care?	☐ Yes	☐ No			
21	If yes, please specify reasons	________________________________________________________				
22	Do oncologists involve you in long-term care of patients?	☐ Consistently	☐ Occasionally	☐ Rarely	☐ Not at all	☐ Varies
23	How would you describe your communication with oncologists?	☐ Very collaborative	☐ Occasional communication	☐ Minimal communication	☐ No communication	☐ Varies
24	In your opinion, how can non-oncologist clinicians contribute more effectively to cancer care?	________________________________________________________				
Additional perspectives						
25	In your view, what is the role of alternative medicine in cancer care? (Ayurveda, Homeopathy, etc.)	☐ Plays an important role, often used	☐ Sometimes supportive	☐ Only patient-driven	☐ No role	☐ Unsure
26	Other observations or suggestions	________________________________________________________				

## References

[1] Sung H, Filho AM, Laversanne M (2026). Global cancer statistics 2024: GLOBOCAN estimates of incidence and mortality worldwide for 34 cancers in 186 countries. CA Cancer J Clin.

[2] Mathur P, Sathishkumar K, Chaturvedi M (2020). Cancer statistics, 2020: report from National Cancer Registry Programme, India. JCO Glob Oncol.

[3] Lingas EC, Ganipisetti VM (2023). Adult hospitalists' knowledge of radiation oncology in an independent nonacademic healthcare system in Albuquerque, New Mexico. Cureus.

[4] Gonçalves M, Gouveia R, Barbosa M, Meireles S (2025). Bridging communication gaps in oncology: insights from a questionnaire among internal medicine and intensive care physicians. Cureus.

[5] Wattana MK, Davenport M, Bischof JJ (2025). Perceived gaps in oncologic emergency care for patients with cancer: a qualitative comparison of emergency medicine and oncologist physician perspectives. Cancers (Basel).

[6] Aemaz Ur Rehman M, Farooq H, Ebaad Ur Rehman M, Ali MM, Zafar A, Khokhar MA (2022). Perceptions of oncology as a career choice among the early career doctors in Pakistan. BMC Med Educ.

[7] Tholeti P, Uppangala S, Jayaram RK (2023). Oncofertility awareness among primary care physicians in India. F1000Res.

[8] Nagar A, Madamanchi D, Nair GR (2024). Barriers to cancer diagnosis and treatment: a pilot qualitative study of patient and practitioner perspectives in rural India. Cureus.

[9] Singh P, Agrawal R (2026). Facilitators and barriers to seeking early diagnosis and treatment for breast cancer among patients attending a tertiary care hospital. Cureus.

[10] Lisy K, Kent J, Dumbrell J, Kelly H, Piper A, Jefford M (2020). Sharing cancer survivorship care between oncology and primary care providers: a qualitative study of health care professionals' experiences. J Clin Med.

[11] Lisy K, Tieu M, Gore C (2025). Oncologist and general practitioner perspectives of shared care for colorectal cancer survivors: a qualitative study. Psychooncology.

[12] Specchia ML, Frisicale EM, Carini E (2020). The impact of tumor board on cancer care: evidence from an umbrella review. BMC Health Serv Res.

[13] Hui D, Bruera E (2020). Models of palliative care delivery for patients with cancer. J Clin Oncol.

[14] Freytag M, Herrlinger U, Hauser S (2020). Higher number of multidisciplinary tumor board meetings per case leads to improved clinical outcome. BMC Cancer.

[15] Siau E, Salazar H, Livergant J, Klein J (2021). Non-oncologist physician knowledge of radiation therapy at an urban community hospital. J Cancer Educ.

[16] Coschi CH, Bainbridge D, Sussman J (2021). Understanding the attitudes and beliefs of oncologists regarding the transitioning and sharing of survivorship care. Curr Oncol.

[17] Piga A, Graziano F, Zahra G, Cellerino R (1996). Attitudes of non-oncology physicians dealing with cancer patients. A survey based on clinical scenarios in Ancona province, central Italy. Tumori.

[18] Shaikh AJ, Khokhar NA, Raza S (2008). Knowledge, attitude and practices of non-oncologist physicians regarding cancer and palliative care: a multi-center study from Pakistan. Asian Pac J Cancer Prev.

[19] Nayar N, Nair R, Mallath MK (2020). Truth telling and fully disclosing to cancer patients in India: a pragmatic approach. Natl Med J India.

